# A case–control study of clinical characteristics and risk factors of symptomatic postpartum pubic symphysis diastasis

**DOI:** 10.1038/s41598-021-82835-8

**Published:** 2021-02-08

**Authors:** Ji-Hee Sung, Mina Kang, Seung-Jae Lim, Suk-Joo Choi, Soo-young Oh, Cheong-Rae Roh

**Affiliations:** 1 Department of Obstetrics and Gynecology, Samsung Medical Center, Sungkyunkwan University School of Medicine, 81 Irwon-ro, Gangnam-gu, Seoul, 06351 Republic of Korea; 2 Department of Orthopedic Surgery, Samsung Medical Center, Sungkyunkwan University School of Medicine, Seoul, Republic of Korea

**Keywords:** Diseases, Risk factors

## Abstract

Postpartum pubic symphysis diastasis (PPSD) refers to the separation of pubic symphysis after delivery. It is typically diagnosed based on clinical symptoms and radiologic findings. This study tried to assess clinical characteristics and risk factors of PPSD. This was a nested case–control study matched for year of delivery and gestational age at delivery using a retrospective cohort of women who delivered vaginally at a single institution. The incidence of PPSD was 0.156% (33/21,131). The incidence rate increased from 0.08% (7/9328) in 2000–2004 to 0.13% (9/7138) in 2005–2009 and to 0.36% (17/4665) in 2010–2016, simultaneously with an increase of maternal age (30.7 ± 3.5 years in 2000–2004 to 31.8 ± 3.8 years in 2005–2009 and 32.8 ± 3.8 years in 2010–2016). Nulliparity was associated with a higher incidence of PPSD (81.8% in cases vs. 57.6% in controls, *p* = 0.01). Other factors including pre-pregnancy body mass index, weight gain during pregnancy, gestational diabetes, induction of labor, duration of labor, epidural anesthesia, vacuum-assisted delivery, episiotomy, neonatal sex and birth weight failed to show difference between the two groups. In short, the incidence of PPSD increased with time along with an increase of maternal age. Nulliparity was the only significant risk factor for PPSD.

## Introduction

Postpartum pubic symphysis diastasis (PPSD) is defined as separation of the pubic symphysis without a fracture after delivery. Although it is an uncommon peripartum complication, it can lead to various problems such as pain, difficulty in ambulation, and urinary dysfunction. Incidence of PPSD has been reported to be 1/30,000 to 1/300 pregnancies with varying degrees^1,2^. In nonpregnant women, the normal gap of symphysis pubis ranges from 4 to 5 mm. During pregnancy under hormonal influence, the symphysis pubis gap increases by at least 2 to 3 mm^3^. If symphyseal separation is more than 1 cm, swelling and inflammation of ligament between pubic bone will occur, resulting in pain and gait difficulty. The pain usually gets worse when bearing a load, moving, or raising legs. Patients often experience radiating pain to the inguinal area, back, and lower extremities. Along with clinical symptoms, imaging methods such as simple X-ray, ultrasonography, computed tomography, and magnetic resonance imaging can be used for making the diagnosis. In most cases, full recovery can be expected within six weeks after conservative treatment including analgesics, bed rest, and pelvic binder. Surgery may be required for extreme cases. However, single-plate fixation is sufficient even for severe cases.

Previous studies have reported that large fetus, small pelvic outlet, multi-parity, operative delivery, previous pelvic trauma, and epidural anesthesia are predisposing factors for PPSD^3,4^. Due to the infrequency of this disease, previous studies are mostly case reports. Little is known about risk factors for PPSD. Thus, the purpose of this study was to determine maternal characteristics and neonatal outcomes associated with PPSD in women who delivered vaginally. Another aim of this study was to evaluate possible risk factors of PPSD by comparing women with PPSD and those without PPSD using tertiary institution data.

## Results

A total of 35,799 pregnancies were reviewed. Of 21,131 women who delivered vaginally, 33 were diagnosed with PPSD. The incidence of PPSD was 0.156% (33/21,131). The median width of symphyseal gap was 2 cm (range, 1.0 to 6.3 cm). Sixteen (48.5%) women had pubic symphyseal widening greater than 2 cm and two women had sacroiliac joint involvement. Among 33 patients with PPSD, five had wide separation greater than 4 cm. All five patients were applied pelvic binder without surgical fixation. One patient who had 5 cm widening of symphysis pubis underwent surgery for urethral injury. However, this patient did not need surgical fixation for PPSD. She was treated with pelvic binder. Although none of these five patients with severe widening of symphyseal gap had gait difficulty, four of them had complaint of persistent pelvic pain at one year postpartum follow up.

For the control group, 132 women with matched year of delivery and gestational age at delivery were randomly selected by incidence density sampling. Maternal characteristics showed no significant difference between the two groups except parity (Table 1). Only nulliparity was associated with a higher incidence of PPSD (81.8% in cases vs. 57.6% in controls, *p* = 0.01). Other factors including pre-pregnancy BMI, weight gain during pregnancy, and gestational or pregestational diabetes mellitus failed to show statistically significant difference between case and control groups. Rates of labor induction, epidural anesthesia, vacuum-assisted delivery, duration of labor, and episiotomy were not significantly different between case and control groups. Shoulder dystocia was observed in two cases in the control group, but none in the case group, and the difference was not statistically significant. Neonatal outcomes were not significantly different between the two groups either (Table 2).

**Table 1:** Maternal characteristics.

	Cases group (n = 33)	Control group (n = 132)	*P* value
**Age (year)**	32.3 ± 4.0	31.1 ± 3.5	0.070
≥ 35	7 (21.2)	19 (14.4)	0.336
< 35	26 (78.8)	113 (85.6)	
**Parity**			
Nulliparous	27 (81.8)	76 (57.6)	0.010
Multiparous	6 (18.2)	56 (42.4)	
Prepregnancy BMI (kg/m^2^)	20.0 ± 2.0	20.4 ± 2.4	0.359
Prepregnancy weight (kg)	51.8 ± 6.9	54.0 ± 6.4	0.082
Weight gain during pregnancy (kg)	13.2 ± 4.2	13.6 ± 4.7	0.650
Height (cm)	160.8 ± 6.2	162.7 ± 4.8	0.062
Gestational or pregestational diabetes mellitus	1 (3.0)	6 (4.5)	1.000

Data expressed as mean ± standard deviation or number (%).

*BMI* body mass index.

**Table 2:** Delivery outcomes.

	Case group (n = 33)	Control group (n = 132)	*P* value
Gestational age at delivery (week)	39.7 ± 1.4	39.7 ± 1.3	0.764
Preterm delivery	2 (6.1)	8 (6.1)	1.000
**Labor induction**	9 (27.3)	35 (26.5)	0.930
Prostaglandin E2	9 (27.3)	33 (25.0)	0.789
Prostaglandin E1	1 (3.0)	5 (3.8)	1.000
Oxytocin augmentation	24 (72.7)	85 (64.4)	0.366
Epidural anesthesia	26 (78.8)	101 (76.5)	0.782
**Delivery mode**			
Spontaneous	30 (90.9)	121 (91.7)	1.000
Vacuum assisted	3 (9.1)	11 (8.3)	
**Episiotomy**	31 (93.9)	122 (92.4)	1.000
No episiotomy	2 (6.1)	10 (7.6)	0.270
Median	15 (45.5)	78 (59.1)	
Mediolateral	16 (48.5)	44 (33.3)	
**Duration of labor**			
First stage (min)	286.6 ± 184.8	269.8 ± 195.8	0.656
Second stage (min)	91.3 ± 79.3	75.4 ± 101.0	0.401
First and second stage (min)	377.9 ± 210.0	345.2 ± 221.8	0.444
**EFW by last ultrasound (g)**	3271.4 ± 416.1	3225.9 ± 396.5	0.561
< 10 percentile	1 (3.0)	4 (3.0)	0.958
10–25 percentile	4 (12.1)	23 (17.4)	
25–50 percentile	10 (30.3)	41 (31.1)	
50–75 percentile	11 (33.3)	44 (33.3)	
75–90 percentile	5 (15.2)	14 (10.6)	
> 90 percentile	2 (6.1)	6 (4.5)	
Shoulder dystocia	0 (0)	2 (1.5)	1.000
Interval between the last ultrasound and delivery (days)	1 (0–23)	1 (0–30)	0.150
Birth weight (g)	3312.1 ± 449.8	3296.2 ± 429.9	0.851
Gender (male)	18 (54.5)	52 (39.4)	0.115
Admission to NICU	2 (6.1)	3 (2.3)	0.261

Data expressed as mean ± standard deviation, median (range) or number (%).

*EFW* estimated fetal weight, *NICU* neonatal intensive care unit.

**Figure 1:**
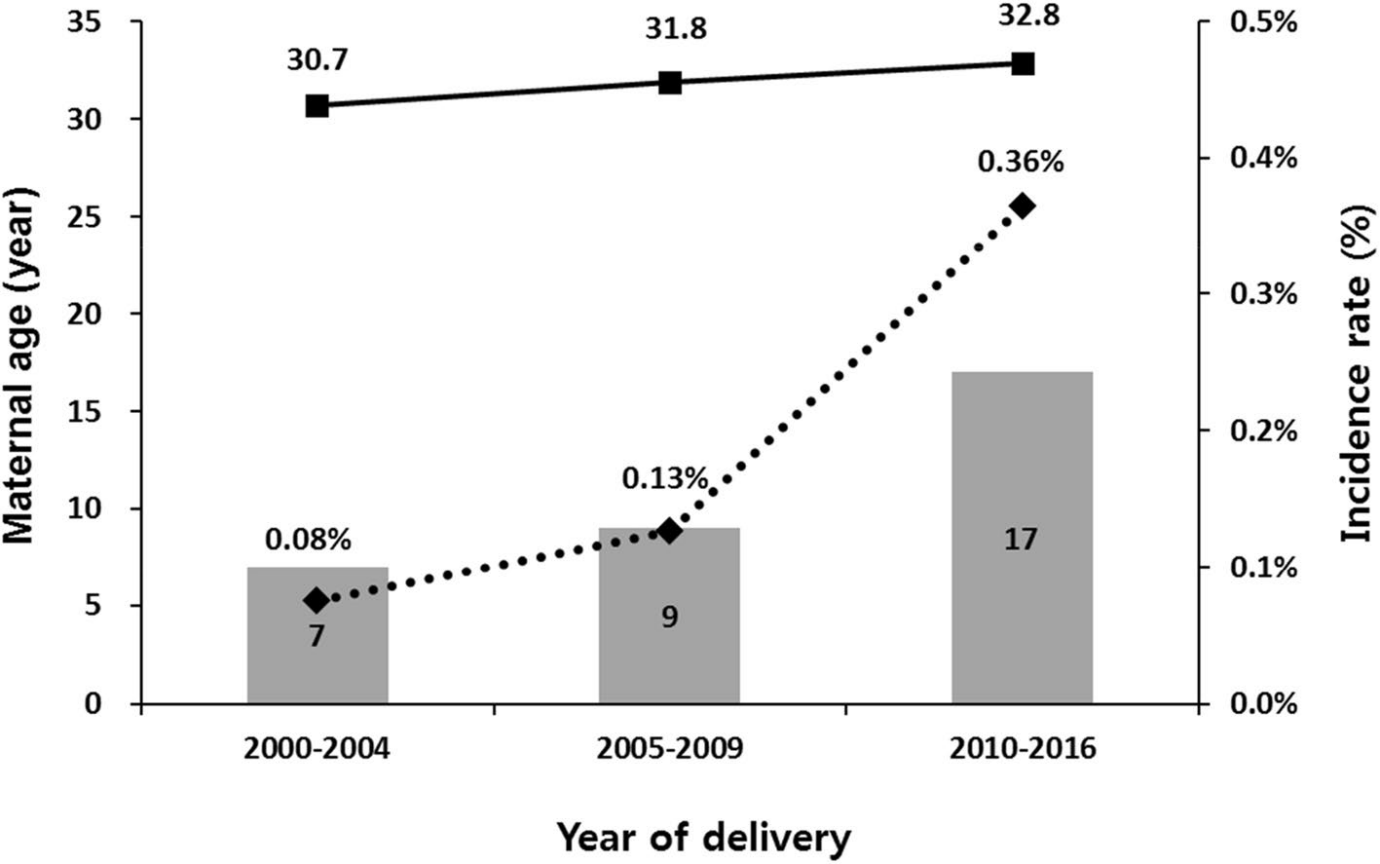
Maternal age (filled black square), number of cases (bar), and incidence rates (filled black diamond) of symphysis pubis diastasis.

Subgroup analysis was conducted to analyze the trend of incidence rate according to study period which was divided into three groups according to the year of delivery: 2000 to 2004, 2005 to 2009, and 2010 to 2016. Incidence rate increased from 0.08% (7/9,328) in 2000–2004 to 0.13% (9/7138) in 2005–2009 and to 0.36% (17/4665) in 2010–2016. Maternal age also increased from 30.7 ± 3.5 years in 2000–2004 to 31.8 ± 3.8 years in 2005–2009 and to 32.8 ± 3.8 years in 2010–2016 (Fig. 1). However, mean maternal age of cases was not significantly higher than that of controls (32.3 ± 4.0 years vs. 31.1 ± 3.5 years, *p* = 0.07).

## Discussion

This study investigated maternal characteristics and risk factors of PPSD in women who delivered vaginally. We found that the incidence of PPSD increased with time along with an increase of maternal age. However, the only statistically significant factor associated with a higher risk of PPSD was nulliparity.

Under the influence of progesterone and relaxin during pregnancy, laxity of joint begins to increase around 10th to 12th weeks of gestation, reaching the maximum at or near term^5^. Accordingly, symphyseal gap is normally widened during pregnancy. It enables women to undergo vaginal delivery^6^. However, if the gap is greater than 1 cm, instability of the symphysis pubis can result in inflammation of joint, leading to ambulatory difficulty. Severe widening of symphysis pubis can damage ligaments linked to pubic bones. If the gap between the symphysis pubis is greater than 2 cm on radiographs, sacroiliac joint involvement is frequently seen. In our study, almost half (16/33, 48.5%) of patients with PPSD had pubic symphyseal widening greater than 2 cm. Of them, two had sacroiliac joint involvement.

**Figure 2:**
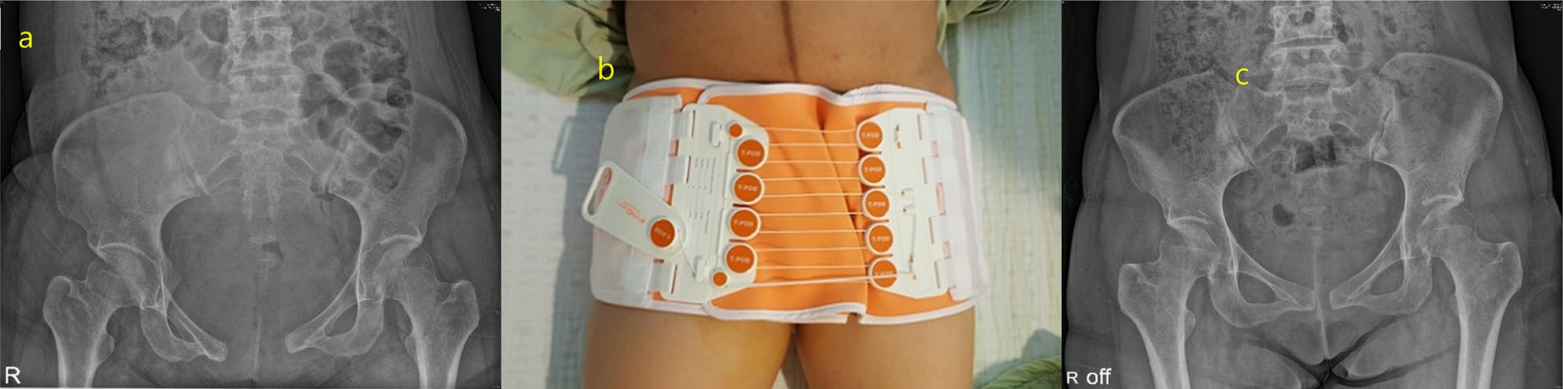
A 34-year-old woman who had pubic pain and gait difficulty after vaginal delivery was diagnosed as PPSD. (**a**) Pelvic radiography at postpartum day 1 showing 5 cm separation of the pubic symphysis. (**b**) She was applied pelvic binder to support pelvic ring integrity. (**c**) Pelvic radiograph at 2-month follow-up showing decreased separation of 1.15 cm.

In most cases, conservative treatments such as bed rest, analgesics, and pelvic binder can be considered as initial treatment options. Nonsteroidal anti-inflammatory drugs and acetaminophen are commonly used for pain control for postpartum women and during breast feeding. Along with adequate analgesics, pelvic binders and pelvic belt or braces are used to support pelvic ring integrity (Fig. 2). However, if conservative treatment fails or symphyseal separation is complicated with urogenital tract trauma, nerve injury, or massive hemorrhage, surgical treatment is indicated. Patients with severe widening of symphyseal gap more than 4 cm could be candidates for surgical fixation^7^. In our study, about 15% of PPSD patients had wide separation greater than 4 cm. One of them underwent surgical treatment for urethral injury. All patients with PPSD in our study were treated with pelvic binder without surgical fixation. The majority (89%) of patients recovered at one year postpartum follow up.

Difficulty in ambulation and sitting can cause physical and emotional discomfort to postpartum women as they experience difficulty in breastfeeding and limitation in taking care of their newborns. Although most patients can recover with conservative treatment, knowing the predisposing factor of PPSD can help us make an early diagnosis of the disease. Along with early detection, immediate establishment of a management strategy may prevent worsening of symptoms of PPSD. As the recurrence rate of PPSD is poorly understood, further studies are needed in the future to inform patients who are planning for subsequent pregnancies.

Although several predisposing factors of PPSD have been reported, the underlying etiology of PPSD is not fully elucidated yet. Multiparity has been reported as one of risk factors of PPSD as progressive weakening of pelvic ligament with each delivery might be related to PPSD^8^. However, this is inconsistent in all cases as PPSD has also been observed in nulliparous women in several studies^2,9^. In 2006, Yoo et al. performed a retrospective cohort study and reviewed 4151 women to investigate risk factors of PPSD. Eleven patients with a diagnosis of PPSD were compared to a control group. They found that nulliparity, multiple gestations, and vaginal delivery were significant risk factors of PPSD^2^. Our study also revealed that nulliparity was associated with a higher risk of PPSD. Although, Yoo et al. investigated a large number of patients, they included women with multiple gestations as well as singleton pregnancies. One of suggested predisposing factors for PPSD is biomechanical strain of pelvic ligaments and associated hyper-lordosis which is more prominent in multiple gestations than in singleton pregnancy. We excluded multiple gestations from our study as pregnant women with multiple gestation would be more prone to experience PPSD than singleton pregnancies. Additionally, we only included women who delivered vaginally because vaginal delivery is a well-known predisposing factor of PPSD and there was no one who had PPSD after cesarean section in our institute.

Women who have small pelvic outlet and those who are pregnant with large fetus are considered to be at high risk of PPSD as pathologic diastasis of pubic symphysis is supposed to be caused by forceful descent of fetus^6,10^. However, this is not a consistent finding because several studies have reported that fetal weight does not play a clear role in the development of PPSD^9,11,12^. A retrospective cohort study has shown that there is no significant difference of neonatal birthweight between the PPSD group and the control group (2.9 ± 0.7 kg in PPSD group vs. 3.0 ± 0.4 kg in control group, *p* = 0.723)^2^. In addition, a recent systematic review of 39 articles has reported that birth weight of newborns in mothers with PPSD varies from 2.8 to 4.8 kg^12^. Our data supported this finding as the rate of large fetus for gestational age and neonatal birth weight failed to show significant differences between the PPSD group and the control group.

Reported incidence of PPSD varies from 1 in 3000 pregnancies to 1 in 300 pregnancies. In 1997, a retrospective review revealed that the incidence of PPSD was close to 1 in 500^10^. A recent study by Yoo et al. has reported that the incidence of PPSD is 1/385^2^. The overall incidence of PPSD in our study was close to 1 in 660. It increased by time from 0.08 to 0.36% during 16 years of study period. Interestingly, maternal age increased by time. This suggests that there might be a positive correlation between the increase of incidence of PPSD and maternal age. However, it is uncertain if this is a true increase of incidence or just a consequence of an increased awareness of the disease. In our study, the mean maternal age of the PPSD group was older than that of the control group (32.3 ± 4.0 years in cases vs. 31.1 ± 3.5 years in controls, *p* = 0.070). The rate of those with advanced maternal age was also higher in the PPSD group compared to that in the control group (21.2% in cases vs. 14.4% in the control, *p* = 0.336), also such differences were not statistically significant. Failure to show statistically significant difference in maternal age between case and control groups might be due to the relatively small size of our study population.

The strength of our study was that the number of cases with PPSD in this study was substantially higher than that in previous reports. To the best of our knowledge, this was a study with the largest number of cases that investigated risk factors of PPSD. Nevertheless, our study might be still underpowered because PPSD is an uncommon disease. Therefore, the sample size was not enough for statistical analysis. Another limitation of this study was its retrospective nature, making it vulnerable to information and misclassification bias. Only symptomatic patients took pelvis X-ray at postpartum period while asymptomatic patients did not have pelvis X-ray results. Although most of PPSD would occur during labor and delivery, some cases might occur before labor. Consequently, asymptomatic pubic diastasis patients might have been included in the control group. Lastly, the generalizability of our study results would be limited because this study was performed in a single tertiary-referral center where most patients were high risk pregnant patients.

In summary, the incidence of PPSD increased with time, suggesting a positive correlation of PPSD with increasing maternal age. The only significant factor that was associated with a higher risk of PPSD between case and control groups was nulliparity.

## Methods

This was a nested case–control study using a retrospective cohort of women who underwent a vaginal delivery of fetus with cephalic presentation at a tertiary hospital from 2000 to 2016. Patients with multiple gestation and those who underwent cesarean section were excluded. Patients who were diagnosed as pubic symphysis diastasis after delivery were selected as cases. All patients who had pain associated with symphyseal separation such as pain at the symphysis pubis after delivery or difficulty in ambulation underwent simple X-ray of pelvis. PPSD was defined as more than 1 cm of symphyseal separation in simple X-ray of pelvis. For each case, four controls matched for year of delivery and gestational age at delivery were randomly selected by incidence density sampling. Maternal characteristics including age, parity, height, pre-pregnancy body weight, pre-pregnancy body mass index (BMI), weight gain during pregnancy, and gestational or pregestational diabetes mellitus were reviewed. All pregnant women underwent a 50 g oral glucose tolerance test as a screening test at 24–28 weeks of gestation or earlier depending on their risk factors. Women who were positive for the screening test underwent a 100 g oral glucose tolerance test and the diagnosis of gestational diabetes mellitus were made using the Carpenter–Coustan criteria. Delivery outcomes included gestational age at delivery, the last estimated fetal weight (EFW) by ultrasonography before delivery, duration of labor, rate of preterm delivery, induction of labor, vacuum-assisted delivery, episiotomy, shoulder dystocia and use of epidural anesthesia. Neonatal outcomes included birthweight, sex, and necessity of admission to neonatal intensive care unit (NICU).

Continuous variables were compared using Mann–Whitney *U* test. Categorical variables were compared using Chi-square test or Fisher’s exact test when one or more expected value was less than 5. A *p* value of less than 0.05 was used to define statistical significance. All tests were two-tailed. All statistical analyses were performed using SPSS version 25 (IBM Corporation, Armonk, NY, USA). This study was approved by the Institutional Review Board (IRB) of Samsung Medical Center (IRB file No. 2020-06-161). Informed consent was exempted by the IRB because this was a retrospective chart review study. All methods were carried out in accordance with relevant guidelines and regulations.

### Ethical approval

This study was approved by the Institutional Review Board for Clinical Research at Samsung Medical Center on July 03, 2020 (IRB No. 2020-06-161).

## Data Availability

Datasets generated during and/or analyzed during the current study are available from the corresponding author upon reasonable request.

## References

[1] Parker JM, Bhattacharjee M (2009). Images in clinical medicine. Peripartum diastasis of the symphysis pubis. N. Engl. J. Med..

[2] Yoo JJ (2014). Incidence and risk factors of symptomatic peripartum diastasis of pubic symphysis. J. Korean Med. Sci..

[3] Nitsche JF, Howell T (2011). Peripartum pubic symphysis separation: A case report and review of the literature. Obstet. Gynecol. Surv..

[4] Kubitz RL, Goodlin RC (1986). Symptomatic separation of the pubic symphysis. South Med. J..

[5] Heckman JD, Sassard R (1994). Musculoskeletal considerations in pregnancy. J. Bone Joint Surg. Am..

[6] Ritchie JR (2003). Orthopedic considerations during pregnancy. Clin. Obstet. Gynecol..

[7] Norvilaite K (2020). Postpartum pubic symphysis diastasis-conservative and surgical treatment methods, incidence of complications: Two case reports and a review of the literature. World J. Clin. Cases.

[8] Bhardwaj A, Nagandla K (2014). Musculoskeletal symptoms and orthopaedic complications in pregnancy: Pathophysiology, diagnostic approaches and modern management. Postgrad. Med. J..

[9] Taylor RN, Sonson RD (1986). Separation of the pubic symphysis: An underrecognized peripartum complication. J. Reprod. Med..

[10] Snow RE, Neubert AG (1997). Peripartum pubic symphysis separation: A case series and review of the literature. Obstet. Gynecol. Surv..

[11] Valsky DV (2006). Postpartum pubic separation associated with prolonged urinary retention following spontaneous delivery. Acta Obstet. Gynecol. Scand..

[12] Urraca-Gesto MA (2015). Diastasis of symphysis pubis and labor: Systematic review. J. Rehabil. Res. Dev..

